# Biomimicking Nature-Inspired Design Structures—An Experimental and Simulation Approach Using Additive Manufacturing

**DOI:** 10.3390/biomimetics7040186

**Published:** 2022-11-03

**Authors:** Arun Y. Patil, Chandrashekhar Hegde, Guruprasad Savanur, Sayed Mohammed Kanakmood, Abhishek M. Contractor, Vinay B. Shirashyad, Rahul M. Chivate, Basavaraj B. Kotturshettar, Shridhar N. Mathad, Mallikarjunagouda B. Patil, Manzoore Elahi M. Soudagar, Islam Md Rizwanul Fattah

**Affiliations:** 1 School of Mechanical Engineering, KLE Technological University, Hubballi 580031, India; 2 Department of Physics, KLE Institute of Technology, Hubballi 580030, India; 3 Bharat Ratna Prof. CNR Rao Research Centre, Basaveshwar Science College, Bagalkot 587101, India; 4 Department of Mechanical Engineering, School of Technology, Glocal University, Delhi-Yamunotri Marg, Saharanpur 247121, India; 5 Department of VLSI Microelectronics, Saveetha School of Engineering, Saveetha Institute of Medical and Technical Sciences, Chennai 602105, India; 6 Centre for Green Technology (CGT), School of Civil and Environmental Engineering, Faculty of Engineering and IT, University of Technology Sydney, Ultimo, NSW 2007, Australia

**Keywords:** nature-inspired architectures, Rhino 7, additive manufacturing, mechanical property improvements

## Abstract

Whether it is a plant- or animal-based bio-inspiration design, it has always been able to address one or more product/component optimisation issues. Today’s scientists or engineers look to nature for an optimal, economically viable, long-term solution. Similarly, a proposal is made in this current work to use seven different bio-inspired structures for automotive impact resistance. All seven of these structures are derived from plant and animal species and are intended to be tested for compressive loading to achieve load-bearing capacity. The work may even cater to optimisation techniques to solve the real-time problem using algorithm-based generative shape designs built using CATIA V6 in unit dimension. The samples were optimised with Rhino 7 software and then simulated with ANSYS workbench. To carry out the comparative study, an experimental work of bioprinting in fused deposition modelling (3D printing) was carried out. The goal is to compare the results across all formats and choose the best-performing concept. The results were obtained for compressive load, flexural load, and fatigue load conditions, particularly the number of life cycles, safety factor, damage tolerance, and bi-axiality indicator. When compared to previous research, the results are in good agreement. Because of their multifunctional properties combining soft and high stiffness and lightweight properties of novel materials, novel materials have many potential applications in the medical, aerospace, and automotive sectors.

## 1. Introduction

Nature is constantly subjected to external noise factors, and it overcomes the variations by tweaking and healing on its own. We, as humans, are always inspired by nature, particularly by things such as living and non-living organisms. There is inspiration drawn from both factors, namely living organisms such as animals, aquatic snails, and plant species, and non-living organisms such as ancient stones, various ores, and so on [1]. In each of these, some unique value propositions are extracted to arrive at an optimal solution to the externally caused effect. Bio-inspirations are transformed into technologies such as bullet trains in locomotion, sustainable buildings based on termite house architecture, bio-decorations focusing on ergonomic and aesthetic designs, self-healing materials in sutures, surfaces derived from shark skin to efficiently overcome deep-sea tides, and adhesion aspects derived from amphibians, to name a few. Recently, bio-inspired design structures are identifying many applications such as aerospace [2,3], the automotive sector [3,4], as well as biomedical fields [5]. Rudraksha, with its scientific name *Elaeocarpus ganitrus*, has unique features to protect its seeds from damage due to mechanical or thermal loads [6]. Rudraksha plants are self-reliant to protect their own seeds along with nutrition and preserve them from early germination of seeds [7]. The structure is efficient enough to behave as a hard shell when external load occurs and, at times, behave as a soft form for opening seed germination [8]. The structural evolution is so unique for Rudraksha that researchers have capitalised on the design form/function in contour building architectures and material applications [9,10,11]. This has made inroads into diversified applications in the science [12,13,14] and engineering [15,16] domains. The tortoiseshell is another such bio-inspiration model that has recently focused on hydrodynamic and strength aspects [17], stiffness of the shell [18], the behaviour of the shell for static and dynamic loading [19], comparison of micro and macro properties [20], performance of rib-suture structure [21], and load distribution of suture mechanism [22,23]. 

Bamboo is another such structure that came to the limelight due to its tensile strength and stiffness [24], along with plenty of fibrous members aligned uniformly [25]. Naturally, composite materials have columnar, porous, well-graded, and lightweight designs [26] that evolved over a million years into bionic structures that can be adopted for structural design. Green buildings and construction are gaining much more importance recently due to their lightweight, low shrinkage rate. Their thermal conductivity is less for bamboo than concrete, sand, and bricks [27]. Storage of carbon, high impact energies, and spectacular mechanical properties are features of green buildings when compared to timber [28]. Green buildings are biodegradable and have a fast rate of growth to maturity [29,30]. Similar to Rudraksha, walnuts are also a useful waste residue left after processing the nuts from the pulp [31]. Walnuts have antioxidant and antidiabetic properties and are preferred in food preservatives [32]. The walnut shell has cellulose 23.9%, hemicellulose 22.4%, 50.1% lignin, and 3.4% ash [33]. More than 50% of lignin will result in a hard-core structure, and a higher cellulose percentage will result in fossil fuel applications [34]. The current work aims to mimic nature-based plants and animals for structures and develop the same with a unit model to arrive at an optimal design. The emphasis is on creating similar structures with the solid modelling tool CATIA V6 and then solving for optimal conditions with the simulation tool ANSYS Workbench. However, to compare experimental and simulation results and validate the results, Rhino 7.1 includes an optimisation tool known as algorithmically generated models. 

Table 1 illustrates the various generic bio-inspired design structures with their mechanical properties studied per ASTM standards. The structures are exemplified with beetle forewing, woodpecker beetle, date palm leaf, corn stalk and reed, Vero white, big sheep horn, hierarchical 3D porous materials, and functionally graded porous bones.

## 2. Background

The primary motivator for this entire project is the use of nature-derived structures/patterns to improve the structural rigidity or robustness of the automotive vehicle’s front, side, or rare region. There were quite a good number of bio-based designs extracted from nature. Among them, the identified design structures are critically well equipped with the strength-to-weight ratio, and long-lasting, sustainable models. These include the Rudraksh, *Bambusa tulda* structure, sheep horn scale, tortoiseshell structure, and walnut structure. etc.

## 3. Why Choose These Patterns?

*Elaeocarpus ganitrus* beads are the dried stones of the fruit of the *Elaeocarpus ganitrus* tree. These beads are hard in physical appearance and have good strength encompassed. These beads have internal hollow portions, with a varying number of hollows in different beads. Altogether this bead can be taken into the study as a bio-inspired model for its characteristics and specifications. When compared to other varieties of wood, it is extremely sturdy and develops quite quickly. *Bambusa tulda* is a fast-growing medium-sized tropical clumping bamboo native to the Indian subcontinent, Indochina, Tibet, and Yunnan. It is a popular and sustainable building material because of its resilience. *Bambusa tulda*’s micro-structure and resulting strength make it the perfect material for bikes and hundreds of other uses. When compared to wood, *Bambusa tulda* fibre is 2–3 times stronger than timber.

Sheep horn serves as the attack and defence weapon during combat. The sheep horn displays amazing mechanical, impact resistance, and energy absorption properties. The ridged pattern we observed on the sheep horn helps promote the properties mentioned above. Thus, we can replicate the sheep horn pattern on a cube and check for various properties such as equivalent stress, total deformation, etc. Tortoiseshell is used as a shield or guard to protect it from the worst climates and other species. The tortoiseshell displays amazing impact resistance and energy/shock absorption properties and acts as the best-covering object. The chip-like structures we observed on the shell help promote the properties mentioned above. Thus, we can replicate the tortoiseshell pattern on a cube and check for various properties such as equivalent stress, total deformation, etc. A *Juglans nigra* is the edible seed of a drupe of any tree of the genus Juglans. It is hard in physical appearance and has good strength. It has a varying number of hollow cross-sections inside it. This can be taken as a good bio-inspired seed for its characteristics and specifications.

## 4. Materials and Methods

**Figure 1:**
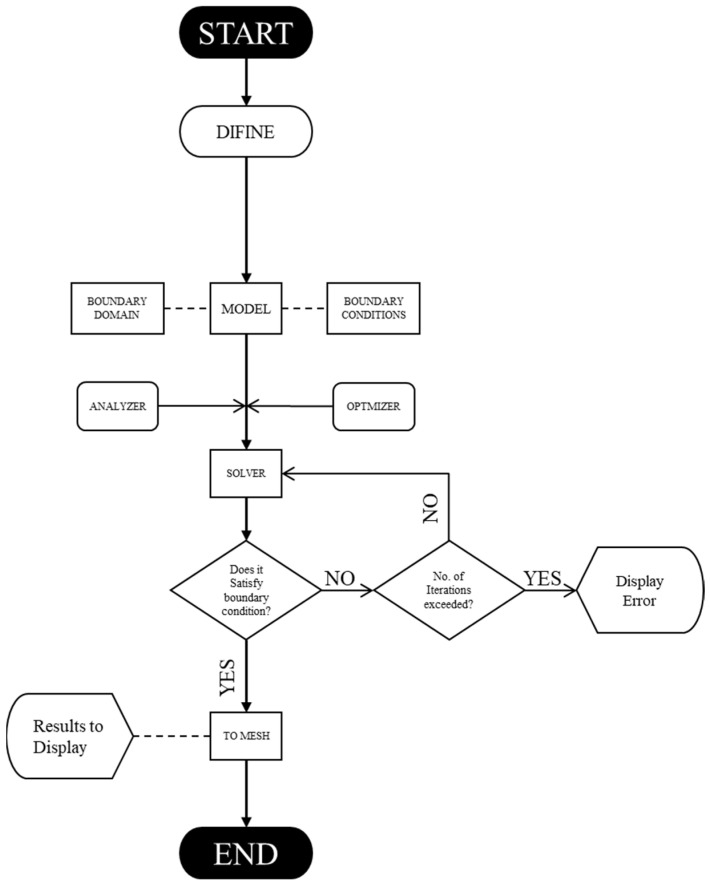
Process map for the flow of optimisation using Rhino 7.

The inspiration to work on crashworthiness for an automotive vehicle derived from two unique areas. Firstly, bio-inspired (nature-inspired) models are extracted from bio species, plant-based species, and even animal scales. Secondly, motivation from the software developed using optimal design methods, such as Altair Hyperworks, inspired solid thinking, Rhino 7 as shown in Figure 1 and Ansys workbench topology optimisation.

### 4.1. Bioinspired Model Selection

**Figure 2:**
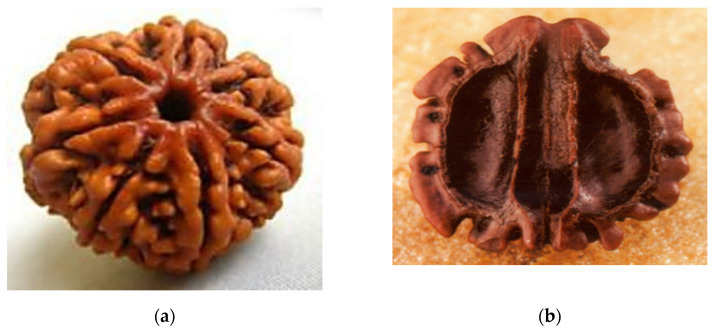

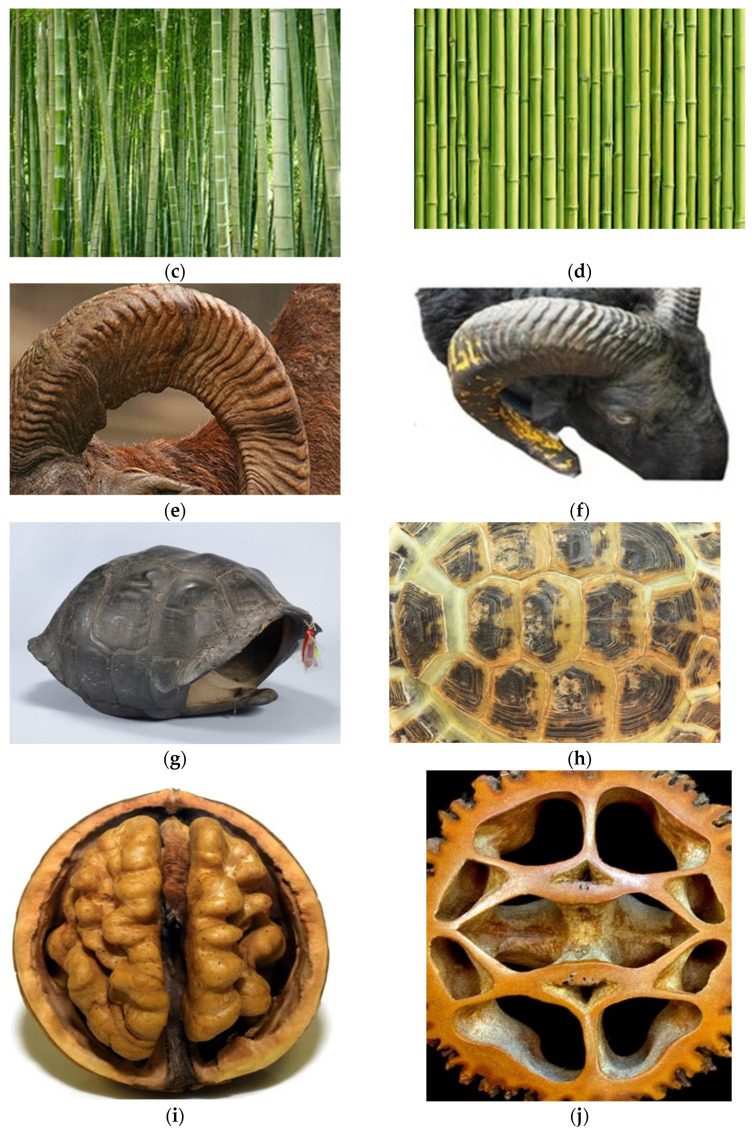
Bio-inspired patterns; (**a**) Rudraksha; (**b**) Rudraksha cross-section; (**c**) *Bambusa tulda*; (**d**) *Bambusa tulda* front view; (**e**) sheep horn outer scale; (**f**) sheep horn outer scale; (**g**) tortoiseshell; (**h**) top view of tortoiseshell; (**i**) walnut; (**j**) walnut cross-section.

The entire literature review has progressed to the point where the identification of new and novel patterns for experimental and simulation study serves as the foundation for future work. The pattern of each identified model has been an inspiration for years since their inception on earth, and authors have clearly distinguished the model with its cross-section, as shown in Figure 2.

### 4.2. Algorithmically Generated Models

#### 4.2.1. Algorithmically Generated Model (AGM)

**Figure 3:**
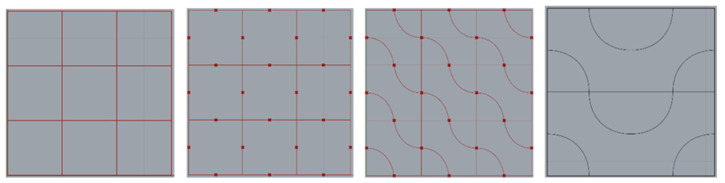
The resulting 3-D geometry was 300 percent upscaled and utilised for testing.

**Figure 4:**
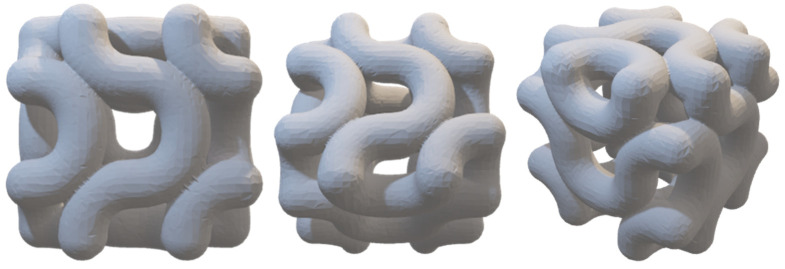
Algorithmically generated model using Rhino 7.

**Figure 5:**
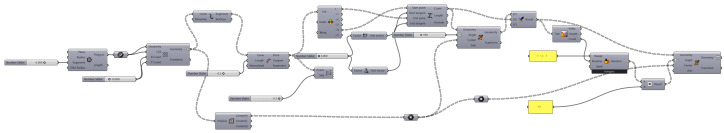
Process workflow in Grasshopper.

Algorithmically generated models (AGMs) are Rhino 7, and Grasshopper was chosen as the software. A 10 mm × 10 mm × 10 mm cube was used, which subsequently split into nine parts of 3 × 3 mm each (Figure 3). Each mini-centre square was linked (Figure 4), and they were given a curvature of 00 to 2700 (Figure 5). This curvature was further changed as needed, with a 30 mm^3^ cube inserted in the surface and the curves given a thickness. The folds of dried instant noodles were the inspiration for this construction. This structure is a straightforward algorithmic manipulation of 2-D geometry.

#### 4.2.2. Bendsoe and Sigmund Optimisation Model

**Figure 6:**
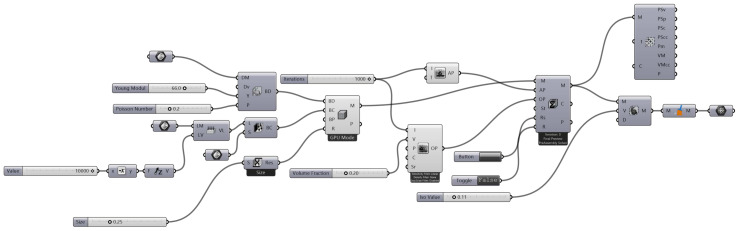
Bendsoe and Sigmund Grasshopper Workflow.

A topology optimisation technique was used to produce this ideal design of an elastic structure. It entails determining the best material distribution in a computational domain that minimises compliance (or, equivalently, optimises stiffness) of the final structure while adhering to a set volume fraction restriction. The Bendsoe and Sigmund Grasshopper Workflow is shown in Figure 6. 

**Figure 7:**
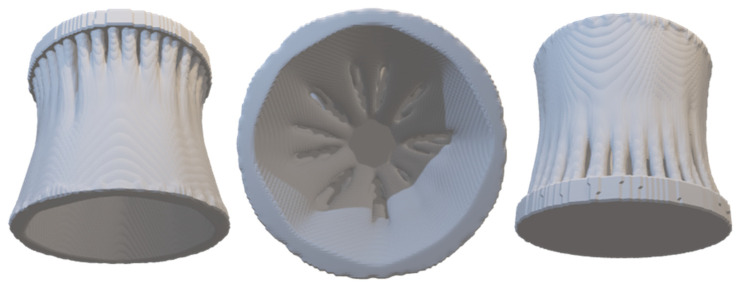
Bendsoe and Sigmund Optimization Model.

A Bendsoe and Sigmund Optimisation on a 2.54 mm (height) and 2.54 mm (diameter) cylinder produced this result. The material used was BSH, and the loading was 100,000 kN under compression, as shown in Figure 7.

#### 4.2.3. Lorimerlite

**Figure 8:**
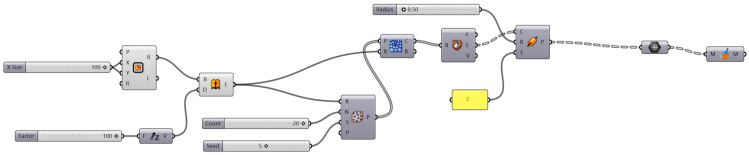
Lorimerlite Grasshopper Workflow.

A topology optimisation technique was used. Show in Figure 8.

## 5. Solid Modelling

This section is divided into subheadings. It provides a concise and precise description of the experimental results, their interpretation, as well as the experimental conclusions that can be drawn.

### 5.1. Elaeocarpus ganitrus (Rudraksha) Model

**Figure 9:**
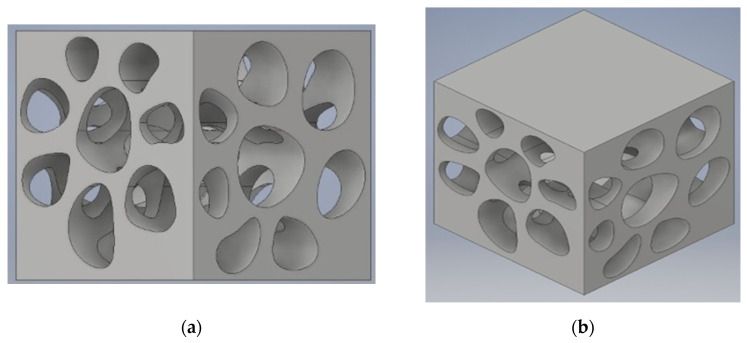
(**a**) *Elaeocarpus ganitrus* model front view; (**b**) Isometric view.

The bio-inspired cross-sectional pattern of this *Elaeocarpus ganitrus* was modelled in Fusion360, and the pattern of 20 × 20 × 20 mm was built. The pattern is shown below in Figure 9a,b in various views.

### 5.2. Bambusa tulda (Indian Bamboo) Model

**Figure 10:**
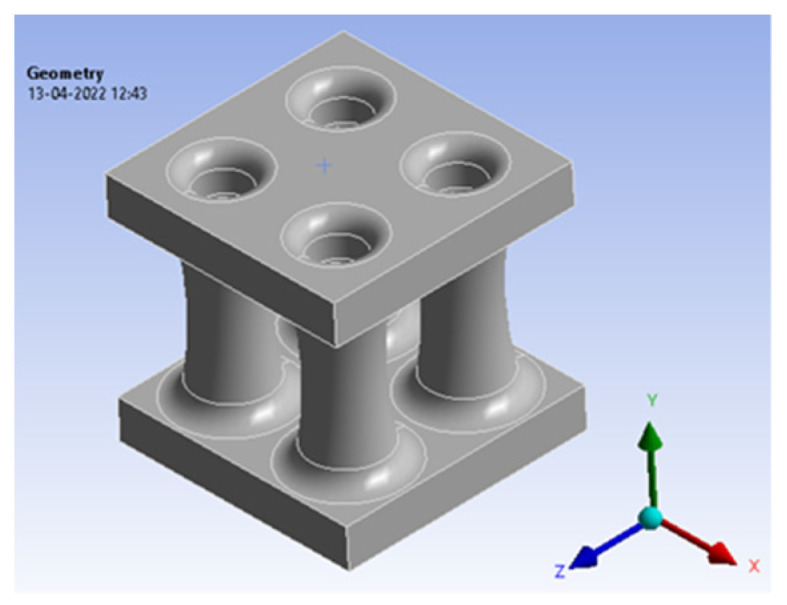
Bambusa tulda model isometric view.

Because of its high tension and compressive strength, *Bambusa tulda* is an excellent reinforcing material. The flexural strength of the beam with *Bambusa tulda* reinforcement is higher, which aids in the better utilisation of *Bambusa tulda*. Tensile strength is greater than compressive strength. The design in Figure 10 was created with Fusion 360 software and measures 30 × 30 × 30 mm.

### 5.3. Sheep Horn Model

**Figure 11:**
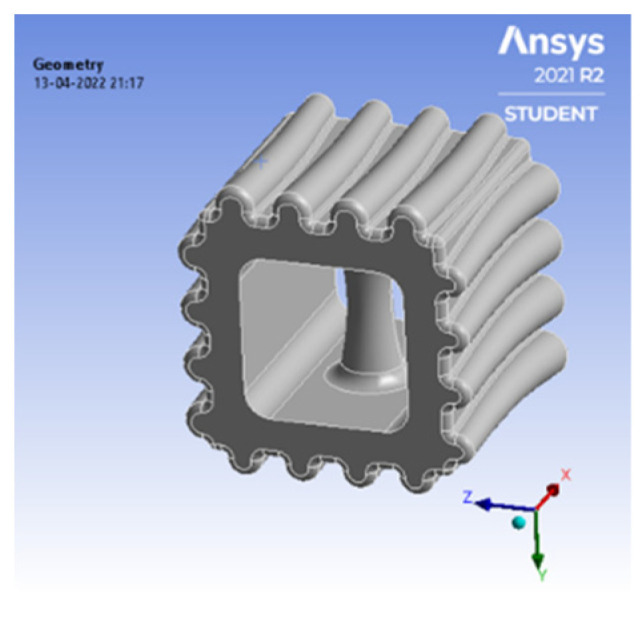
Sheep horn scale.

A 3D model of the cube was rendered, and the sheep horn pattern was embossed via CATIA 3D experience software. The pattern, as shown in Figure 11 is made up of 20 × 20 × 20 mm.

### 5.4. Tortoiseshell

**Figure 12:**
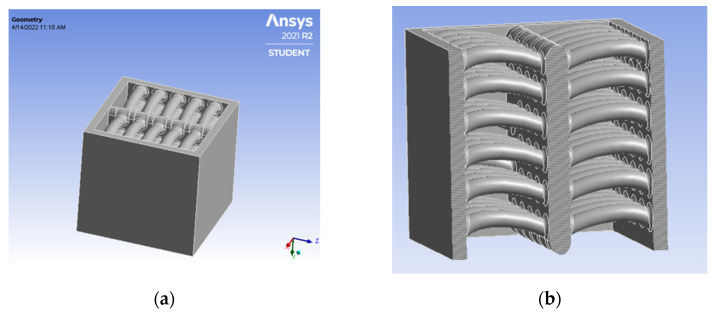
(**a**) Tortoiseshell isometric view; (**b**) Cross-sectional view.

A 3D model of the cube was rendered, and the sheep horn pattern was embossed via Fusion 360 software. Figure 12 was built with a 20 × 20 × 20 mm size.

### 5.5. Juglans nigra (Walnut)

**Figure 13:**
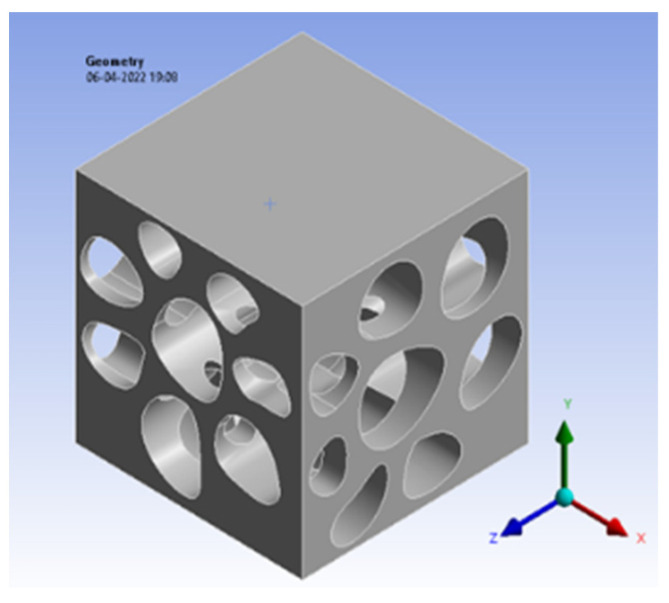
Walnut model view.

The bio-inspired cross-section pattern of the *Juglans nigra* is modelled in Fusion 360, and the pattern is built with a size of 20 × 20 × 20 mm. The pattern is shown below in Figure 13.

## 6. Simulation Study

The simulation of the bio-inspired pattern was carried out using Ansys Workbench. The simulation was carried out for two different materials, i.e., structural steel and sheep horn. A load of 10,000 N was applied on top of the structure, and the base of the cube was fixed in all six degrees of freedom. 

### 6.1. Elaeocarpus ganitrus Model

**Figure 14:**
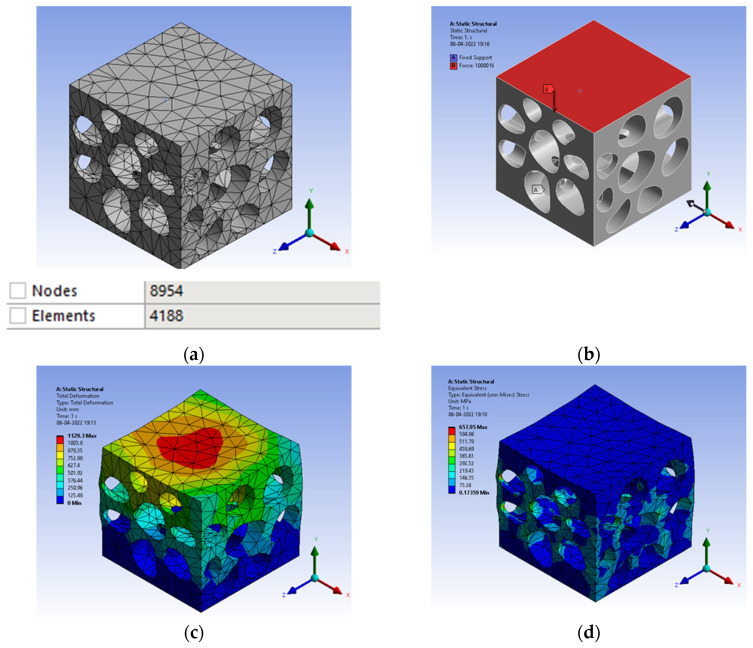
(**a**) Mesh generation; (**b**) Loads and boundary conditions; (**c**) Total deformation; (**d**) Von mises stress.

The bio-inspiration from *Elaeocarpus ganitrus* showed a lot of prominence in the structural application as the load in compression resulted in von mises stress of 657 MPa, as shown in Figure 14b. Mesh carried out with tetrahedron element made with ‘Solid 92’, a 10-noded elemental model, as shown in Figure 14a. Figure 14a infer nodes 8954 and elements 4188 were appropriate for the simulation. The element size with the H-type method is preferred to arrive at the convergence of the solution. The total deformation shows a slightly higher condition of deformation compared to the solid model. Figure 14c has the total deformation and Figure 14d deals with stress results. 

### 6.2. Bambusa tulda Model

**Figure 15:**
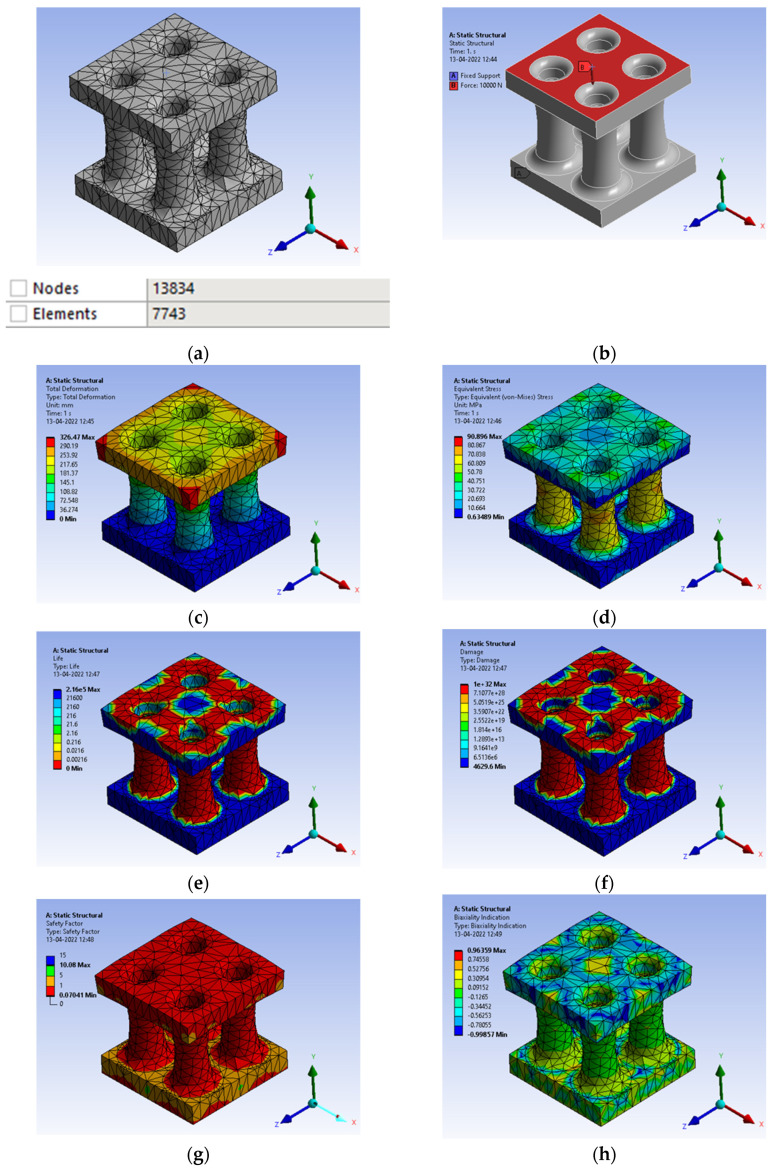
(**a**) Mesh generation; (**b**) Loads and boundary conditions; (**c**) Total deformation; (**d**) Von mises stress; (**e**) Life cycle; (**f**) Damage; (**g**) Factor of safety; (**h**) Biaxiality indication.

Bamboo or *bambusa tulda* is an inspiration in nature for its structural property, such as tensile strength and oxygen filter model, and has equal potential to replace the Thermo Mechanically Treated (TMT) rod in concrete columns. However, when it comes to simulation comparison, it does fit well as the replacement for structural applications, showing enormous potential with 90 MPa in Figure 15d as lower than the yield value of 110 MPa. Further, fatigue analysis is carried out for identification of the number of cycles as shown in Figure 15e for the stress-based approach with mean stress Goodman modified theory showing 2.16 × 10^5^ cycles. The mesh generation shows nodes 13834 and elements 7743, resulting in a convergence solution. A load of 10,000 N is acting in compressive form with a fixed support at the bottom surface. A total deformation of 326.47 mm was observed in Figure 15c. 

### 6.3. Sheep Horn Model

**Figure 16:**
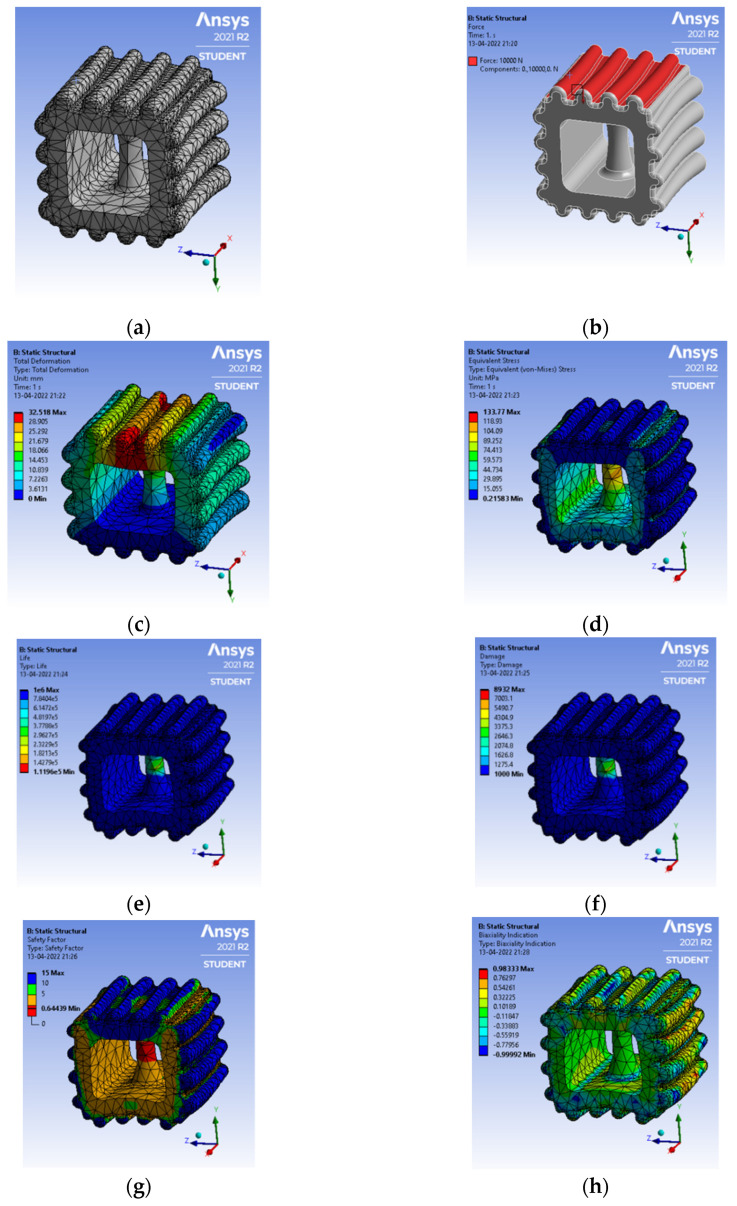
(**a**) Mesh generation; (**b**) Loads and boundary conditions; (**c**) Total deformation; (**d**) Von mises stress; (**e**) Life cycle; (**f**) Damage; (**g**) Factor of safety; (**h**) Biaxiality indication.

Sheep horn outer skin mimicked the structure of a typical kind, as shown in Figure 16a, with mesh generation of a tetrahedron element, and the load applied on top of the structure results in a total deformation of 32 mm and von mises stress of 133.7 MPa, as shown in Figure 16c, d, respectively. The outer structure mimicked the horn’s outer skin with an embossing [stiffener] kind of structure. Figure 16e shows the life cycle as 1.11 × 10^5^, slightly higher than any typical component’s design life cycle. The factor of safety shows as 0.64 but needs to be in the range of 1.1 to 1.5.

### 6.4. Tortoise Model

**Figure 17:**
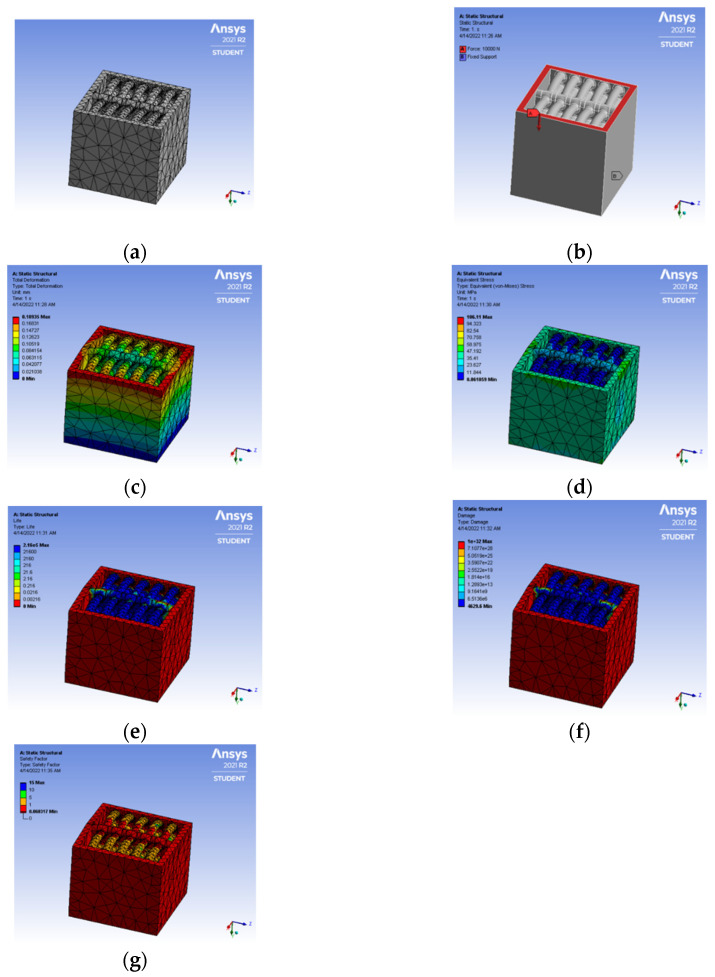
(**a**) Mesh generation; (**b**) Loads and boundary conditions; (**c**) Total deformation; (**d**) Von mises stress; (**e**) Life cycle; (**f**) Damage; (**g**) Factor of safety.

The tortoiseshell structure has been a benchmarked model when it comes to biomimicking nature-based species. The uniqueness of the tortoiseshell structure is it builds an unconventional design structure between the carapace to vertebrae and ribs. This typical structure is built in the CATIA model and converted into neutral file format .stp for further analysis in the ANSYS workbench. In Figure 17a–g, the entire process of simulation is depicted, with as low as 0.18 mm total deformation and von mises stress of 106 MPa. The material is safe in terms of the ultimate criteria of Polylactic acid (PLA) for 3D printing conditions. The life cycle of the member is typically above the design life cycle criteria, i.e., 2.16 × 10^5^ cycles.

### 6.5. Juglans regia (Walnut) Model

**Figure 18:**
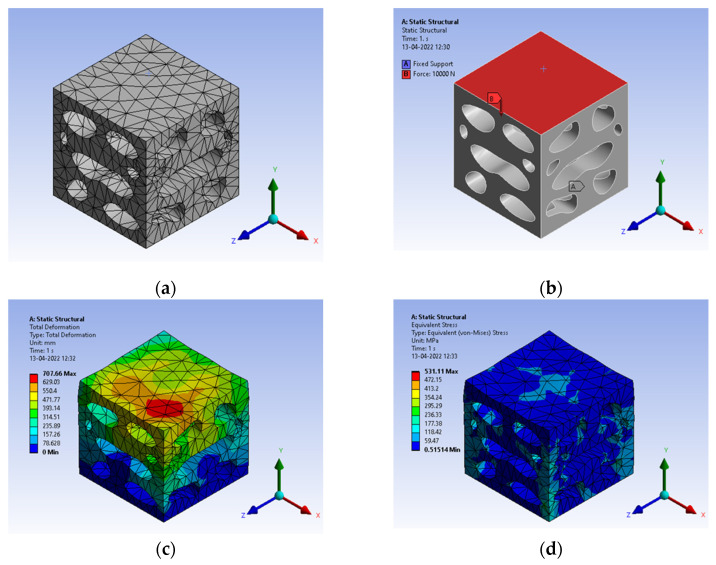
(**a**) Mesh generation; (**b**) Loads and boundary conditions; (**c**) Total deformation; (**d**) Von mises stress.

Another plant-based species with a unique structure and highly impact resistive model is the walnut. Juglans regia is versatile in its structure due to the pericarp to endocarp structure transformation because of internal shape and size decided by walnut seed forms. Once the walnut is extracted from the endocarp, the internal structure is mimicked for its highly compressive and impact-resistant application. In Figure 18a–d, the entire process of simulation is carried out to arrive at outcomes with total deformation observed of 707 mm and von mises stress of 472 MPa, which is higher in value for its design criteria limit. 

## 7. Experimental Work

### 7.1. Algorithmatically Generated Models (AGM)

**Figure 19:**
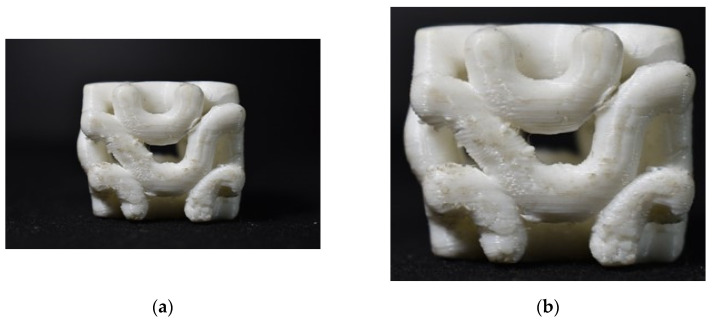
(**a**) A 3D printed AGM; (**b**) Tested model with failed structure.

**Figure 20:**
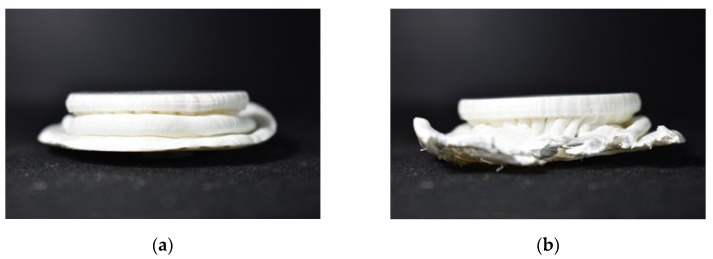
(**a**) Compressed model; (**b**) Failed model while testing.

The work is partially fulfilled if experimental aspects are not dealt with in the course of the research activity. The AGM and Bendsoe and Sigmund models have been able to withstand the highest ultimate load for a typical 3D printed structure using the Rhino 7 software model. Figure 19a is inferred as a 3D printed model with 1293 lbf load-bearing capacity for ultimate force. The observations drawn in Figure 20a are the Bendsoe and Sigmund models, and have resulted in an ultimate force load-bearing capacity of 2222 lbf, which is the highest among all the cases.

### 7.2. Bio-Inspired Models

**Figure 21:**
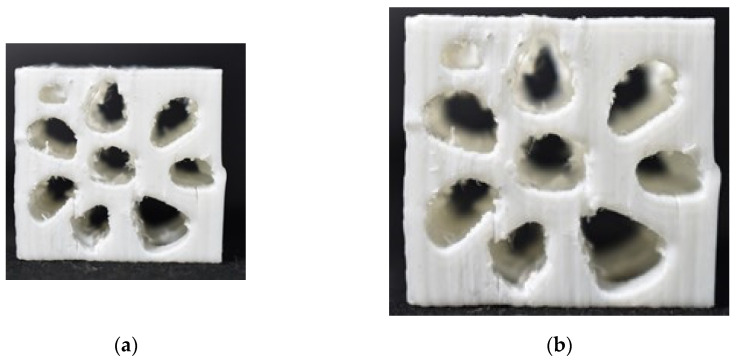

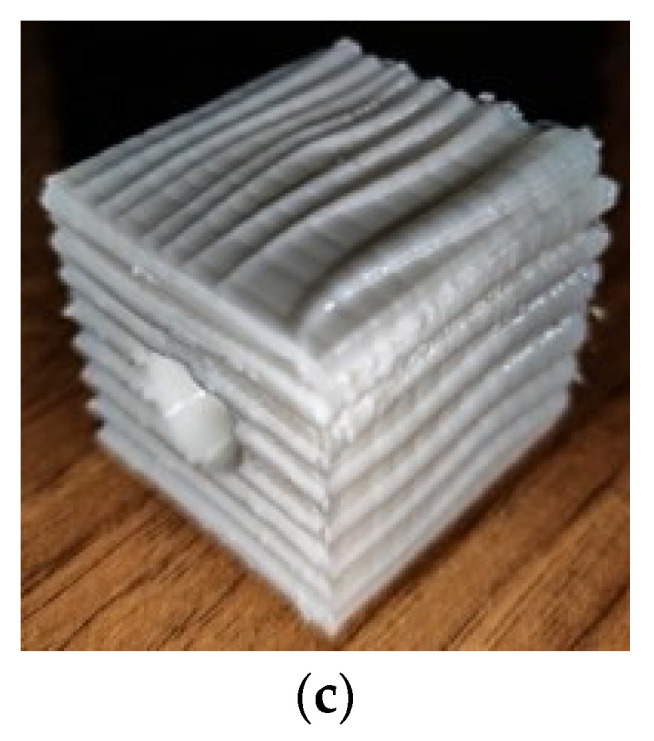
(**a**) Compressed Rudraksha model; (**b**) Failed Rudraksha model; (**c**) Horn mimic model.

A typical case of the walnut structure with a 3D printed model is illustrated in Figure 21a, and adjacent to that Figure 21b shows a compression-tested and failed model of the same 3D printed model.

## 8. Results and Discussion

The current work focused on eight different bio-inspired patterns, and each design structure was subjected to a number of iterations to arrive at an optimal design model. The entire result section does a comparative study with respect to mechanical strength, as shown in Table 2. In the entire study, the Bendsoe and Sigmund model has equivalent load-bearing capacity compared to the 50% filled standard model.

Furthermore, the study covers the simulation study for mechanical strength, as illustrated in Table 3. These seven kinds of bio-inspired patterns are compared for total deformation, von mises and maximum principal stress theory, fatigue life cycle, and factor of safety calculation using the ANSYS workbench software tool. Among all the five bio-inspired patterns, the tortoiseshell has the lowest total deformation of 0.189 mm and maximum principal stress of 106 MPa, resulting in the most optimal model. Table 4 depicts the bamboo structure, Table 5 the Rudraksha, Table 6 the horn embossing pattern, Table 7 the tortoise vertebral structure, and Table 8 the walnut structure for three types of material study structural steel, sheep horn, and PLA. The sheep horn and structural steel material-based study revealed fatigue life cycle and factor of safety. The investigation carried out for Rudraksha with 1800–3000 N of compressive load resulted in higher specific strength in comparison to brick, concrete, and porcelain [53]. However, the shell observed with high Vickers hardness (210 ± 30 MPa) differentiated with the commercial aluminum (1100–0, annealed). Based on the observation with Rudraksha, various other shell structures such as walnuts, hazelnuts, pecans, and almonds were later investigated. In addition, the shell described above is thought to have five times lower compressive loads than Rudraksha [54,55]. However, the highest mechanical properties observed for Rudraksha have been reported to arise through numerous distinct structural features, such as highly lignified and multiple shapes of sclereids [56,57,58]. Furthermore, in the case of bamboo culms subjected to axial compressive and tangential load for a three-case failure loading of high, medium, and low conditions. The obtained results show ~99 ± 5 MPa, ~101 ± 5 Mpa, and ~100 ± 5 MPa, respectively. However, tangential loading has ~17 ± 1 MPa, ~20 ± 1 MPa, and ~16 ± 1 MPa, respectively. The results are in close agreement with Awalluddin [59]. Onche [60] developed a compressive strength model of bamboo using the empirical relation of fleck and budiansky [61]. The work focused on the modelling failure analysis of composites. Korde and west [62] depicted the kinking and the fibre buckling of fibre within a band when subjected to compressive load.

## 9. Conclusions

The study on seven bio-inspired models for automotive impact resistive application has resulted in the following outcomes:

➢ Development of a unique process map for optimization model using the Rhino 7 software tool to realize the least material condition logic in geometric dimensioning and tolerancing.
➢ Creation of a database for seven bio-inspired models for the typical mechanical property extraction such as tensile strength, flexural strength, fatigue behaviour parameters viz, life cycle, the factor of safety, damage tolerance, and bi-axiality indication.
➢ Production of complex geometrical shapes using an experimental method known as fuse deposition modelling (FDM) and arriving at the outcomes to further compare experimental results with simulation results and earlier data to draw a conclusion.
➢ Generation of design methods in the future in terms of designing and optimizing 3D complex models, as well as conducting a combined parametric and topology optimization scheme.
➢ The maximum principal stresses for Rudraksh, *Bambusa tulda*, sheep horn, tortoiseshell, and *Juglans nigra* are 657.95 MPa, 90.89 MPa, 145.44 MPa, 106.11 MPa, and 531.11 MPa, respectively, and their fatigue life cycles were 2.16 × 10^5^, 2.16 × 10^5^, 1 × 10^6^, 1 × 10^6^ and 2.16 × 10^5^, respectively.

## Figures and Tables

**Table 1:** Bio-inspired design models built by predecessor authors.

Sl No	Title	Author and Year	Description (Test Conducted)	Pattern Images
1	Bio-inspired columns mimicking beetle forewing structure (review paper)	Xiang and Du [35], 2017	The specific energy absorption of the bio-inspired multi-cell tubes (BMTs) with a length of 50 mm, a velocity of 10 m/s, and a mass of 500 kg increased by 9.79 percent and 35.97 percent, respectively, as compared to the standard A velocity of 10 m/s for impact. When compared to the traditional structure, the Specific Energy Absorption (SEA) of the BMTs (b), (c), and (d) rose by 90.56 percent, 68.33 percent, and 107.68 percent, respectively (a).	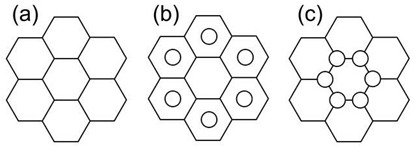
		Hao and Du [36], 2018	The length and diameter of the columns were 200 mm and 377 mm, respectively, and absorption properties improved at a velocity of 10 m/s and a mass of 500 kg.	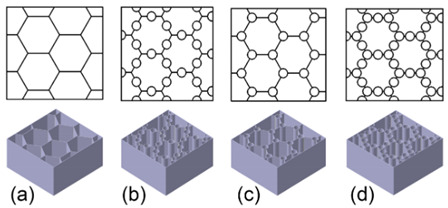
		Xiang et al. [37], 2017	The specific energy absorption of the bio-inspired multi-cell tubes (BMTs) with a length of 50 mm, a velocity of 10 m/s, and a mass of 500 kg increased by 9.79 percent and 35.97 percent, respectively, as compared to the standard A velocity of 10 m/s for impact. When compared to the traditional structure, the Specific Energy Absorption (SEA) of the BMTs rose by 90.56 percent, 68.33 percent, and 107.68 percent, respectively (a).	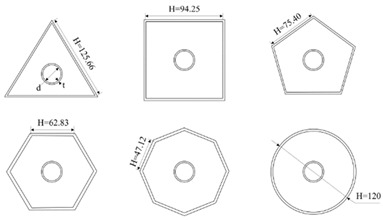
		Yu et al. [38], 2018	The SEA of the bio-inspired hexagonal structures was 5× that of a corrugated square box.	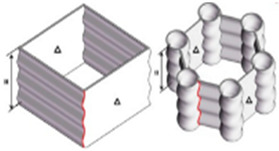
2	Bio-inspired multi-cell conical tube mimicking ox horn structure.	Meng et al. [39], 2015	The SEA of the bio-inspired tube was 46.2 kJ/kg, which was about 1.3 times and 1.8 times greater than that of four cell conical tube and conical tubes.	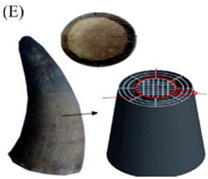
3	Microstructure of a woodpecker’s beak to design a novel BSP	Ha et al. [40], 2019	The SEA increased by 125% and 63.7%.	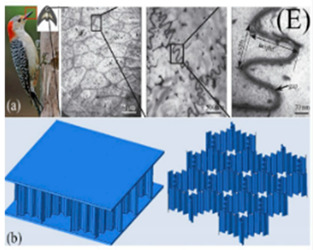
4	Date palm leaf fibre (DPLF)	Mahdi et al. [41], 2019	The SEA of DPLF unidirectional reinforced epoxy composites rectangular tubes was 10.3 kJ/kg.	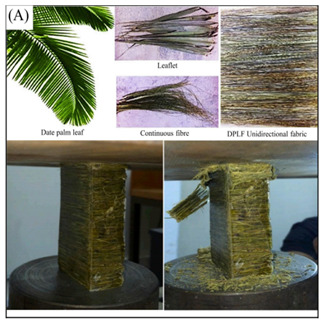
5	On the crashworthiness of bio-inspired hexagonal prismatic tubes under axial compression	Wang [42], 2020	Bio-inspired hexagonal prismatic tubes with a height of 120 mm and with different interior cell arrangements, with a velocity of 10 m/s impacted a plate and a tube. Increasing the SEA about 2.5 times the normal hexagonal prism. This study also shows variation in EA and axial compression with respect to geometrical structures of the same and different weights.	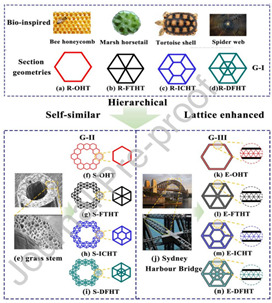
6	Experimental study on the crashworthiness of bio-inspired aluminum foam-filled tubes under axial compression loading	Jiafeng Song [43], 2020	Based on two typical straw structures (Cornstalk and Reed), four types of bio-inspired foam-filled thin-walled structures were created.	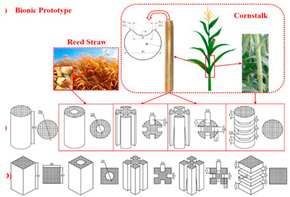
7	Separating the influence of the cortex and foam on the mechanical properties of porcupine quills	Wen Yang, Joanna McKittrick [44], 2013	The experimental value of the Buckling strength of the Hystrix structure with cortices is 135.2 + 29.6 MPa or 135.2−29.6 MPa and the theoretical value is 83.9 MPa (for E = 2.6 GPa). Simiar to the Experimental Buckling strength of the Erethizon is 19.9 + 8.5 MPa or 19.9 − 8.5 MPa and the theoretical value is 26 MPa (for E = 1 GPa). The modulus for the Hystrix cortex is 2.6 times higher than for the Erethizon cortex (1 GPa).	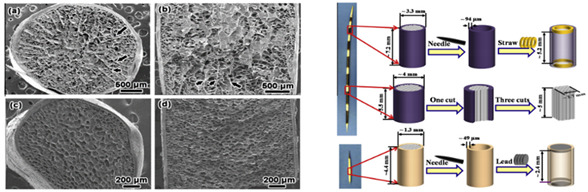
8	Bio-inspired energy-absorbing material designs using additive manufacturing	Aniket Ingrole [45], 2021	(1) Vero White Plus (VW) photopolymer Soft Matrix-rubberlike digital substance D9860-Abolone sheep horn. RESULTS static and dynamic testing on polymer composites demonstrated a specific loss modulus of up to 0.43 km/s^2^, which is the greatest of any damping material. The energy absorption capacities of the structure increased by 25% and 120 percent in drop impact testing.	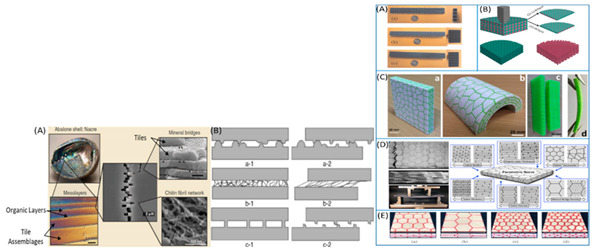
			(2) Conch shell-additive manufacturing was employed, and the materials tested were Veromagenta Stiff and Tango BlackPlus. When compared to a single-level hierarchy and a rigid constituent, a second-level hierarchy structure improves impact performance by 70% and 85%, respectively.	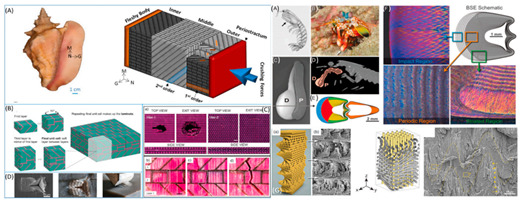
			(3) Dactyl club-TangoBlackPlus for Bouligand and Herringbone matrixes The results indicated a 3.4-fold increase in energy absorption between the herringbone (1.20 Jm^−3^) and Bouligand structures when using VeroWhitePlus for the fibres (0.35 Jm^−3^). The orientation and distribution of the fibres affect the composites’ impact, toughness, and compression strength.	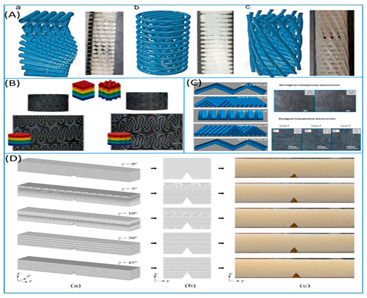
			(4) Stiff material for horns and hooves specimens with hexagonal prisms placed at 0–22.5–45 angles had a greater critical stress intensity factor and a higher critical energy release rate, according to the SENB method: FORCE OF IMPACT: 3400 N for horns and 9000 N for hooves.	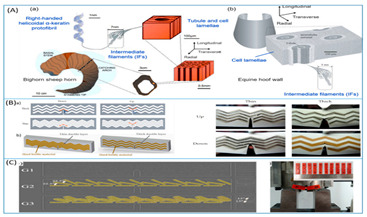
			(5) Beetle electron-DSM Somos 14120 resin compression testing to determine the structure’s deformation modes and energy absorption capability. BEP has a higher compressive strength (15%), deformation (63%), and energy absorption (115%) than HP, which has a specific energy absorption of 9.16 103 J/kg and an energy absorption of 154.80 J.	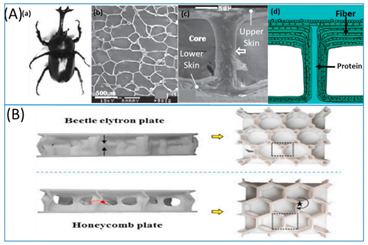
9	Hierarchical structure and compressive deformation mechanisms of bighorn sheep (Ovis canadensis) horn	Wei Huang [46], 2017	Impact force = 3400 N, velocity 9 m/s strain rate of order-10^2^–10^3^. Young’s modules 0.65 to 2.2 GPa for X-ray-size 2 × 2 × 4 mm^3^. From impact area, impact area thickness 2 cm, inner thickness 1 cm. Optical and scanning electron microscopy imaging and compression test-size-4 × 4 × 4 mm^3^, 5 mm from import surface. Transmission electron microscopy imaging -size 2 × 1 × 1 mm^3^. Young’s modulus increases in the day state 2–3 times when the strain rate changes from 1 × 10^3^/s to 4 × 10^3^/s. Radial direction (impact direction) was found to have the highest strength and energy absorption, inspired by a design of recoverable energy-absorbing material.	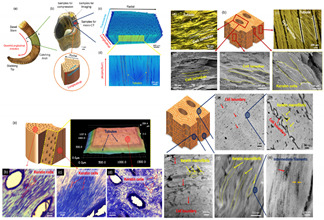
10	Design optimisation of a novel bio-inspired 3D porous structure for crashworthiness	Hanfeng Yin [47], 2020	Four types of H3DP [Hierarchical 3D porous] porous material are popular types of materials and are lightweight, have excellent sound absorption, and have high impact energy absorption. Based on the quasistatic simulation, structures with different lattice configurations ranging from 4 × 4 × 4 to 7 × 7 × 7 tessellating cells are investigated. An Instron 8802 universal test machine is used to perform the quasistatic compression test on the H3DP structure. The bottom plate simply supports the lattice samples, while the upper platform is crushed at a rate of 0.2 mm/min.	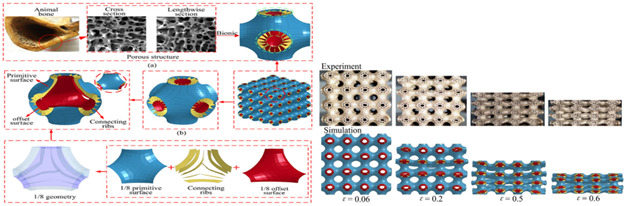
11	Energy absorption of bio-inspired multi-layered graded foam-filled structures under axial crushing	Xinmei Xiang [48], 2020	Model-1 with graded foam filler exhibited larger SEA (specific energy absorption). Model-1 (250-400-550) is considered the best design for the foam-filled structures investigated in this study. Values: EA = 2590.2 J, CFE = 66%.	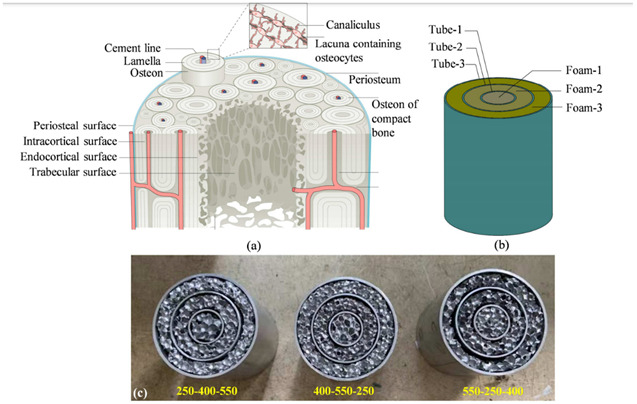
12	A review of the impact-resistant biological and bioinspired materials and structures	Benjamin S. Lazarus [49], 2020	Keratin scutes easily delaminate and deflect cracks, toughening the shell. Young’s modulus of these layers are about 20 GPa and 1 GPa, respectively. On the human skull, shear punch tests were performed at strain rates of 0.001 s^−1^ and 0.1 s^−1^. The skull of a woodpecker. Conch shells dissipate energy as well by causing cracks to meander. The conch shell has a 67 percent higher fracture strength when tested at a strain rate of 103 s^−1^ than when tested at a strain rate of 104 s^−1^. The third-order lamellae fracture and splinter away under impact loading.	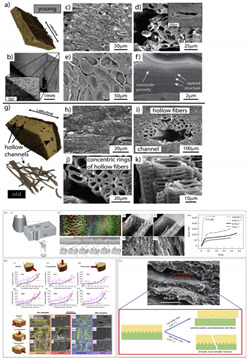
13	Experimental and numerical assessment of sustainable bamboo core sandwich panels under low-velocity impact	Lívia Ávila de Oliveir [50], 2021	Sandwich panels were subjected to a drop test with a 16 mm diameter hemispherical impactor. A mass of 9.46 kg is dropped at 3.25 m/s on the sandwich panels. A finite element model of the drop test simulation was also conducted at 20-Epoxy-Max load (kN) = 4.5, total deflection (mm) = 22.80, 20-Biopolymer-Max load (kN) = 6.29, and total deflection(mm) = 11.49.	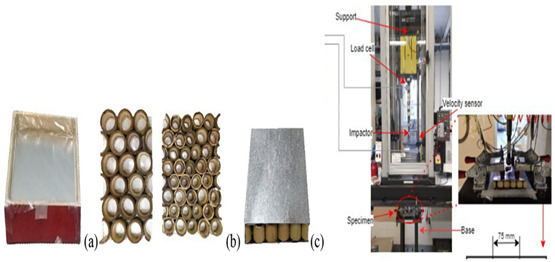
14	Laser additive manufacturing of bio-inspired lattice structure. Forming quality, microstructure, and energy absorption behaviour	Yuexin Du [51], 2019	AlSi10Mg alloy powder was used as the raw material in this study, Fmax (2.95 kN) with a displacement value (1.18 mm) at 375 W. As the laser power increased to 400 W or 450 W, the accumulated displacement drastically decreased. At the end of stage II, the force dropped sharply accompanying the failure of a certain strut occurred.	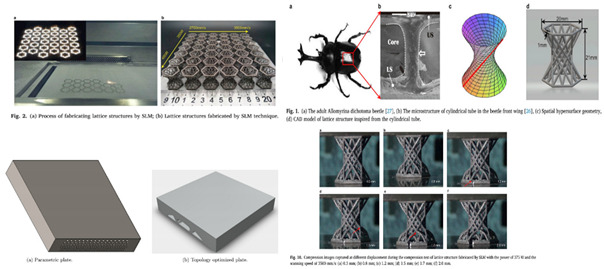
15	Through-thickness perforated steel plates optimised for ballistic impact applications	Francisco Javier Ramírez-Gil [52], 2021	(1) The weight of steel plates is reduced by introducing holes through the thickness. (2) Two different design methodologies are followed: heuristic and systematic. (3) The heuristic method uses bioinspired principles of functionally graded porous bones.	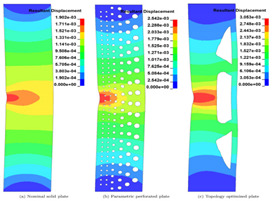

**Table 2:** Generic comparison of a few cases for mechanical strength.

Pattern	Ultimate Force (lbf)	Ultimate Stress (psi)
Standard (50% fill)	2381.12	2173.28
Rudraksh	789.13	720.25
AGM	1293.39	-
Bendsoe and Sigmund model	2222.42	707.42

**Table 3:** Simulation study comparison for sheep horn material.

Pattern	Total Deformation (mm)	Maximum Principal Stress (MPa)	Fatigue Life Cycle	Factor of Safety
Rudraksh	1129.3	657.95	2.16 × 10^5^	0.09
*Bambusa tulda*	326.47	90.89	2.16 × 10^5^	0.07
Sheep horn	0.338	145.44	1 × 10^6^	0.593
Tortoiseshell	0.189	106.11	1 × 10^6^	0.058
*Juglans nigra*	707.66	531.11	2.16 × 10^5^	0.009

**Table 4:** Simulation study comparison for bamboo structure.

Type	Material		
	Structural Steel	Sheep Horn	PLA
Total deformation	0.010273 mm	326.47 mm	0.68835 mm
Equivalent stress	92.83 mm	90.896 MPa	95.387
Fatigue life	Max − 1 × 10^6^ Min − 0	Max − 2.16 × 10^5^ Min − 0	
Fatigue Damage	1532.1	1 × 10^32^	
Fatigue safety factor	Max 15 Min 0.92857	Max 10.08 Min 0.07041	
Biaxiality indication	Max 0.97225 Min − 0.97544	Max 0.96359 Min − 0.99996	

**Table 5:** Simulation study comparison for Rudraksha structure.

Results	Structural Steel	Sheep Horn	PLA
Total Deformation	0.036271 mm	1129.3 mm	2.6932 mm
Equivalent Stress	659.08 MPa	657.95 MPa	1049.4 MPa
Life	1 × 10^6^ − Max 703.46 − Min	2.16 × 10^5^ − Max 0 − Min	
Damage	1.42 × 10^6^ − Max 1000 − Min	1 × 10^32^ − Max 4629.6 − Min	
Safety	15 − Max 0 − Min	15 − Max 0 − Min	
Biaxiality Indication	0.988 − Max −0.99 − Min	0.98822 − Max − 0.99967 − Min	

**Table 6:** Simulation study comparison for horn structure.

Results	Structural Steel	Sheep Horn	PLA
Total deformation (mm)	0.011245	0.33809	0.711
Equivalent stress (Mpa)	130.7	145.44	172.49
Strain Energy (mJ)	0.43981	13.714	
Life	Max: 1 × 10^6^ Min: 1.281 × 10^5^	Max: 1 × 10^6^ Min: 82,488	
Damage	Max: 8209.6 Min: 1000	Max: 12,133 Min: 1000	
Safety factor	Max: 15 Min: 0	Max: 15 Min: 0	
Biaxiality Indicator	Max: 0.9859 Min: − 0.999	Max: 0.98333 Min: −0.999	

**Table 7:** Simulation study comparison for tortoise vertebral structure.

Results	Sheep Horn	Structural Steel	PLA
Total deformation (mm)	0.18936	6.1907 × 10^−6^	0.41234
Equivalent stress (MPa)	106.11	1.2653 × 10^8^	89.819
Life	Max: 2.16 × 10^5^ Min: 0	1 × 10^6^ 1.3698 × 10^5^	
Damage	Max: 1 × 10^32^ Min: 4629.6	7300.1 1000	
Safety factor	Max: 15 Min: 0.06	15 0.6	

**Table 8:** Simulation study comparison for walnut structure.

Results	Sheep Horn	Structural Steel	PLA
Total deformation (mm)	0.034741 mm	0.08722 mm	2.3286 mm
Equivalent stress (MPa)	1680.1 MPa	1424.5 MPa	1690.6 MPa
Life	Max 1 × 10^6^ Min (0 cycles)	Max 0 Min 0	
Damage	1.5041 × 10^7^	1 × 10^32^	
Safety factor	Max 15	Max 15	

## Data Availability

Not applicable.
